# Telemedicine‐medical “snack community”‐PHS ecosystem: Insights into the double‐edged sword role of telemedicine in clinical practice and medical education during the COVID‐19 pandemic and beyond

**DOI:** 10.1002/EXP.20230111

**Published:** 2024-01-23

**Authors:** Dechao Feng, Xu Shi, Jie Wang, Liying Zhang, Yuhan Xiao, Dengxiong Li, Ruicheng Wu, Wuran Wei, Akira Miyamoto, Koo Han Yoo, Xing Ye, Chi Zhang, Ping Han

**Affiliations:** ^1^ Department of Urology, Institute of Urology West China Hospital, Sichuan University Chengdu China; ^2^ West China School of medicine West China Hospital Sichuan University Chengdu China; ^3^ Department of Rehabilitation The Affiliated Hospital of Southwest Medical University Luzhou P. R. China; ^4^ Department of Rehabilitation West Kyushu University Fukuoka Japan; ^5^ Department of Urology Kyung Hee University Seoul South Korea; ^6^ Cedars‐Sinai Medical Center Los Angeles California USA

**Keywords:** clinical surgery, medical education, partners healthcare system, telemedicine

## Abstract

Telemedicine has gained tremendous development during the COVID‐19 pandemic. With deblocking and opening, telemedicine accelerates the evolvution of the medical “snack community” and undermines the perception of medical students and staff, which promotes the incidence of psychosocial‐related disorders. Moreover, the inconsistent telemedicine adaptability between medical workers and patients aggravates the doctor–patient conflict due to the aging population and COVID‐19 squeal. Telemedicine is colliding with the national healthcare system, whose synchronization with conventional medical service is crucial to coordinate the relationship among medical payment, patient privacy and qualifications of clinicians. This study puts more emphasis on the double‐edged sword role of telemedicine in clinical practice and medical education during the COVID‐19 pandemic and beyond. Overall, while telemedicine has demonstrated its utility in health care throughout the COVID pandemic, it is pretty critical to continue evaluating the efficacy and limitations of telemedicine in order to maintain equal access to medical service and high‐quality medical education. A new concept as telemedicine‐medical “snack community”‐PHS ecosystem, where the psychological health education system and partners healthcare system with enough bandwidth, especially 5G technology, could optimize the effect of telemedicine on medical practice and education, is proposed.

## Graphical abstract

1. “Telemedicine‐medical ‘snack community’‐PHS ecosystem” indicates the plight during the transformation of traditional culture into sack culture in modern society.
2. The supports of regulations made by government and the third‐party supervision are prerequisite for medical education and fitness of medical practitioners and patients.
3. Constructions of infrastructure, medical education and expertise of medical workers are far more important.

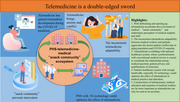

## 1 BACKGROUND

Telemedicine or telehealth is not a novel concept and get a lot more attention until 2019, as can be seen from the annual publications on PubMed. There is no doubt that COVID‐19 prevention policy is an all‐important catalyst that contributes to the dramatic development of telemedicine, primarily focusing on real‐time, intense and comprehensive patients’ management via advanced electronic systems and 5G technology, which is quite important for post‐surgery rehabilitation or isolated patients at home.^[^ ^1^, ^2^, ^3^, ^4^, ^5^ ^]^ “Internet +” refers to the use of information and Internet platforms to integrate the Internet with traditional industries and then use the advantages of the Internet to create new development opportunities. With the help of “Internet+,” compared to traditional offline service, telemedicine can almost complete most medical services through online platforms or application software, such as consultation, ward rounds, video conferencing, patient education, psychological support, examination and evaluation, health monitoring, and so on. Unfortunately, telemedicine is responsible for reshaping a new era of medical “snack community” and is affecting everyone in their daily lives, especially with deblocking and opening up worldwide. In this condition, we put more emphasis on the double‐edged sword role of telemedicine in clinical practice and medical education during the COVID‐19 pandemic and beyond.

## 2 MEDICAL “SNACK COMMUNITY”

Snack culture is a kind of cultural trend and cultural phenomenon based on the accelerated rhythm of society that pursues quick, popular, and short‐term popularity. This is due to the convenience and cheapness of spiritual fulfillment, which takes away the motivation to explore the unknown and reduces people's tolerance for pain because innovation means more time and effort, which is painful. The current snack culture is eroding modern society, including medical practitioners and medical students, who are defined as medical “snack community.” Medical “snack community” refers to those who prioritize efficiency, speed, and quick pleasure over thorough analysis in the medical field. These people may have the following characteristics: (1) Overreliance on quick solutions: They might be more likely to rely on standardized medical choices and treatment protocols rather than performing individualized assessments. (2) Technology‐driven tool dependence: Rather than depending on their own clinical expertise and judgment, they can tend to rely on health information systems and applications. (3) Potentially jeopardized doctor‐patient bond: They can be under time pressure at work to decide quickly and pay less attention to a thorough examination of the situation. Prioritizing efficiency and speed over interpersonal relationships and communication with patients might have unintended consequences. Additionally, we supposed that the phenomenon of the medical “snack community” was accelerated by the rapid development of telemedicine throughout the COVID‐19 pandemic.

The mechanism behind the medical “snack community” shows the dilemma during the transformation of traditional culture in modern society, which is thought‐provoking. The popularity of snack culture has to a certain extent relieved the pressure o “snack community,” but it has brought negative effects on the majority of “snack community” in terms of the construction of value consciousness, moral orientation and language thinking abilities as well as clinical and research abilities. For clinical practitioners, those “snack community” simply uses the convenience brought by technology and equipment, and is not willing to think about the principles behind it and has difficulty detecting complex diseases and potential diseases. Therefore, they are gradually reduced to “essay work machines,” and this part of the “snack community” group may be replaced by artificial intelligence today.^[^ ^6^, ^7^, ^8^ ^]^ Moreover, neglecting the improvement of one's own clinical skills and experience also leads to a reduced capacity for self‐renewal.

In terms of medical students, joining the “snack community” will lead to unexercised medical thinking and reduced innovation. The first and foremost of these is the ability to read. The “snack community” type of reading often does not seek to understand and does not spend a lot of time and energy to explore and summarize in depth. Easier anxiety is also one of the characteristics of “snack community.” The decline in their creative thinking and the ease of mental gratification makes the “snack community” not only loses their ability to explore, but also reduce their tolerance for pain and their ability to deal with it. In the era of the medical “snack community,” both medical practitioners and students tend to have ‘brain burnout syndrome’. It has the following features: (1) Broad and superficial thinking: Individuals may become excessively sidetracked and fixate on an excessive amount of unimportant or unimportant information, which results in superficial and naive thinking. This can make it more difficult to solve problems and make decisions since it is hard to concentrate on significant concerns. (2) Mechanical repetition: Instead of doing original or creative work, individuals could tend to complete mechanically repetitious duties at work or school. Their inclination to repeat things without much thought may have inhibited their creativity and mental activity. (3) Diminished cognitive function: They might have a reduction in executive and cognitive performance. Specifically, they might experience mental exhaustion, struggle to stay focused, experience memory loss, and struggle with logical thought and problem solving. As a result, people can perform worse at work or school because they are having cognitive challenges. To sum up, “brain burnout syndrome” is a condition of cognitive exhaustion characterized by shallow thinking, high repetition and noticeably reduced cognitive function. Students and healthcare workers may experience it, particularly if they are under a lot of pressure to study or work for extended periods of time, which can be accelerated by a telemedicine‐mediated fast‐paced lifestyle. Over time, these populations are more prone to experience anxiety, boredom, job burnout, depression, and other psychological and physical illnesses, which will negatively impact both their quality of life and the patients’ medical outcomes.

The responses from doctors and medical students could be classified as nonclinical (like COVID‐related policy and research and supporting vulnerable groups in the community), clinical (like remote clinical care and triage, assistance in COVID testing or treatment centers, and contact tracing), and educational (like making materials to inform peers, the community, or community health workers, and specialty society meetings) during the COVID‐19 pandemic.^[^ ^9^ ^]^ Telemedicine refers to “the provision of health care services over a spatial distance through the use of telecommunication technology with the aim of benefiting a patient or population.”^[^ ^10^ ^]^ It can save per‐person health care expenditures while also improving patient outcomes and health.^[^ ^11^ ^]^ Telemedicine has expanded at an exponential rate in recent years. Its applications encompass surgery (telesurgery), telerehabilitation, a remote intensive care unit, and chronic disease management, outdistancing phone triage and ambulatory electronic visits (e‐visits).^[^ ^12^ ^]^ Even before the epidemic, it was estimated that the telemedicine business would expand from $38.3 billion in 2018 to $130.5 billion by 2025.^[^ ^13^ ^]^ Moreover, hurdles to its broad usage during the COVID‐19 pandemic were swiftly crumbling, and patients appeared to welcome it.^[^ ^14^ ^]^ Self‐reported disadvantages of telemedicine from medical students and clinicians during the pandemic included patient safety and privacy, efficiency of care, and the potential for medical errors.^[^ ^15^ ^]^ Due to the lockdown measures, many districts banned outdoor as well as classroom activities. It could be challenging for practitioners who have never utilized telemedicine to adapt their usual interview and physical exam techniques to the virtual world.^[^ ^16^ ^]^ The barriers to telemedicine application included patient discomfort, possible technological illiteracy, and the stress associated with adapting to a new environment or system, which contributed to the increase of the medical “snack community.”^[^ ^16^ ^]^ In addition to the sudden change in the traditional face‐to‐face model, medical “snack community” is facing high levels of academic pressure.^[^ ^17^ ^]^ As the famous saying goes, “Publish or perish!” Firstly, although telemedicine virtually allows for many postponed or cancelled medical meetings of specialty societies, offering versatility and time‐saving opportunities for busy clinicians and medical students, the number and frequency of scientific webinars increased significantly, which made it more difficult for them to control their virtual agenda under the COVID‐19 pandemic and in turn increased the number of medical “snack communities” and the subsequent adverse chain reaction.^[^ ^18^ ^]^ At this point, this will also lead to a feeling of digital burnout for such a population due to a lack of supervision and time management. Secondly, the COVID‐19 pandemic outbreak has increased the professional burden of health care workers, accompanied by financial stress, resulting in an increased number of medical “snack communities,” decreased satisfaction and quality of life, and more anxiety.^[^ ^19^ ^]^ Thirdly, health care workers, especially in the medical “snack community” is more likely to occur in times of epidemic burnout due to increased work hours, reduced personal fulfillment, and emotional exhaustion, which might be exacerbated by the slather of telemedicine and further increase the formation of medical “snack community” and a series of subsequent consequences.^[^ ^20^ ^]^

Additionally, telemedicine also exerts an impact on traditional medical education as traditional clerkships have been suspended at medical schools and most courses are offered online, especially for practice‐based courses.^[^ ^21^, ^22^ ^]^ Telemedicine may allow medical students to restart their studies while still maintaining a safe atmosphere by integrating multiple entrustable professional activities for entering residency to ensure a uniform set of skills for all medical school graduates.^[^ ^13^ ^]^ Incorporating telemedicine into medical school curricula would not only expose students to pertinent technology but also enhance their comprehension of the intricate ethical, regulatory, and legal considerations that accompany these cases.^[^ ^12^ ^]^ Although most studies have reported benefits of online education, including managing time and expertise presenting,^[^ ^22^, ^23^ ^]^ it can be challenging for medical students to deal with extraneous cognition in an online environment, such as extraneous and redundant sounds, words, or visuals such as images, which cause distractions for learners.^[^ ^24^ ^]^ Additionally, interactions between students and providers are more distant when conducted through a computer screen or phone. Patient safety issues and inconsistent care quality might result from inadequate or disorganized efforts to offer high‐quality telemedicine education. Additionally, it can add to the already heavy workload that medical students already have.^[^ ^12^ ^]^ When traditional classroom or in‐person instruction gives way to tele‐delivered curriculum, environmental factors that affect students from vulnerable, underprivileged, or minority backgrounds include unemployment of the students themselves and their family members, unequal access to and provision of educational technologies and remote delivery platforms, and elevated levels of stress on mental health due to extended periods of isolation and self‐quarantine.^[^ ^25^ ^]^ Importantly, the above concerns might lead to the increment of medical “snack community.” Another result of medical “snack community” lower self‐restraint is the addiction of smartphones common in telemedicine, which works in conjunction with anxiety and can also lead to insomnia.^[^ ^26^, ^27^, ^28^ ^]^ COVID‐19 also brought increased levels of depression and anxiety to the student population.^[^ ^29^ ^]^ Among them, central and bridging symptoms may be the core symptoms that trigger or maintain the development of anxiety and depression among university students.^[^ ^30^ ^]^ Anxiety generation is partly due to the “fast food” culture that leads to the “snack community” preferring to accept simple visuals and images and refusing to learn difficult textual information, which is necessary for medical learning. In addition, the lack of teamwork opportunities and lack of face‐to‐face communication opportunities may also lead to anxiety and other psychological related disorders.^[^ ^31^ ^]^ This even includes daily infection precautions at work, reducing social etiquette habits such as shaking hands and maintaining social distance, which were found to lead to boredom, anxiety, and depression.^[^ ^32^, ^33^ ^]^ An interesting finding is that the rise of the take‐out industry, especially during the pandemic, has led to a reluctance of the younger generation to go out and forage for food, as “snack community” also tends to opt for fast food in their diet and sedentary lifestyle, as well as a lack of physical exercise. This indirect effect of the pandemic led to an increase in fat mass in “snack community” due to the consumption of energy‐dense foods. This increased fat content was also positively correlated with stress, which is detrimental to physical and mental health.^[^ ^34^, ^35^ ^]^

In the post‐epidemic era, the shift from online to offline teaching was also a blow to medical “snack community.” The researchers found that the change from online courses to on‐site learning in the post‐COVID‐19 pandemic era affected students’ stress levels, causing them to develop stress disorders.^[^ ^36^ ^]^ This increased stress will have harmful effects on both physical and mental health outcomes, leading to psychiatric symptoms such as cognitive dysfunction, dementia, and excessive fatigue, while reducing physical activity.^[^ ^37^ ^]^ And chronic stress can affect dietary intake and increase vasoconstriction, leading to hypertension and left ventricular hypertrophy.^[^ ^34^, ^38^ ^]^ Furthermore, for medical “snack community” who are not adept at coping with elevated work intensity and difficulty and are called to work in hospitals to compensate for manpower shortages that cause them to face time allocation pressures, Liu et al. reported the prevalence of fatigue symptoms among Chinese nursing students.^[^ ^31^ ^]^ The lower adaptive capacity of medical “snack community” makes it more likely that this fatigue symptom will translate into anxiety and depression, or even lead to an imbalance in the adjustment/recovery system.^[^ ^39^, ^40^ ^]^ In addition, pandemic is caused by the severe economic form, affecting the employment situation, more medical students are forced to choose to go on to higher education. This competitive pressure will inevitably aggravate “snack community” anxiety, depression and other psychological disorders, due to its poorer ability to resist stress. Notably, medical “snack community” for telemedicine produced decreased perception also partly attributed to COVID‐19 infection. Folayan et al. found that a history of COVID‐19 infection was associated with memory problems as well as emotional disturbances.^[^ ^41^ ^]^ This also suggests to us that raising awareness and thinking positively during a pandemic is extremely important and necessary for medical “snack community.”

## 3 INCONSISTENCE OF DOCTOR‐PATIENT TELEMEDICINE ADAPTABILITY

In the past 3 years, the COVID‐19 pandemic has had a significant impact on people's production and living in China and beyond. Not only have many COVID‐19 patients been unable to receive adequate medical care, but patients with other diseases can't access their regular medical care due to the squeeze of medical resources. In this context, the rapid development of telemedicine with its unique technological advantages and convenience has helped to alleviate the imbalance of medical resources to a certain extent. However, with deblocking and opening up in China, the contradictions and challenges brought by telemedicine have come to the fore recently.

For patients, medical workers have built‐in advantages over patients in telemedicine technologies. They possess more medical knowledge and skills, making it easier for them to adapt to and master telemedicine technologies. In contrast, patients may have various levels of adaptability to telemedicine due to age, education, and geographic differences. The difference in telemedicine adaptability between medical workers and patients is inevitable. In addition, an aging population might contribute to the development of telemedicine adaptability. By 2030, ≈20% of the world's population will be aged 65 or older.^[^ ^42^ ^]^ According to the National Bureau of Statistics of China, by the end of 2019, the population aged 60 or above had reached 254 million, accounting for 18.1% of the total population.^[^ ^43^ ^]^ Furthermore, as of June 2020, the 46th Statistical Report on Internet Development in China indicated that around 96 million mobile Internet users were aged 60 and above, which meant that over 150 million elderly people did not use the Internet.^[^ ^44^ ^]^ In fact, less telemedicine was used among older adults, racial and ethnic minorities, people with lower educational attainment and people with lower socioeconomic status, and the COVID‐19 pandemic further exacerbated this situation, which was called “digital divide.”^[^ ^45^, ^46^ ^]^ Moreover, the development of telemedicine adaptability is limited by the imbalance between the convenience of telemedicine and its quality and technology limitations, such as fewer medical services, an unstable video network environment, non‐standard information and privacy management and so on. It is worth noting that during the COVID‐19 pandemic, the older adults were more inclined to in‐person medical visits than telemedicine.^[^ ^47^ ^]^ Of the 208 participants who completed the questionnaire, 39.5% thought telemedicine visits were worse than in‐person medical visits and only 4.9% thought they were better.^[^ ^47^ ^]^

For medical workers, the adaptability to telemedicine varies among individuals. The heterogeneity among the medical workers exerts a significant challenge to the development of telemedicine adaptability. On the one hand, in‐person medical visits already occupy the majority of their time and energy for some specialists and professors, especially in tertiary hospitals. For example, in China's top hospitals, each doctor needs to deal with a large number of patients every day, and if they are in university affiliated hospitals, they also have to undertake some teaching and research tasks. This makes it difficult for them to attend a telemedicine consultation. On the other hand, for some ordinary doctors in underdeveloped areas, the “threshold” of telemedicine software and hardware may deter them. Firstly, telemedicine requires doctors to master various communication and monitoring technologies, such as video conferencing, remote monitoring equipment, internet and data security, but necessary training and guidance for these technologies may not be effectively implemented for them. Secondly, the differences in hardware conditions, such as anesthesia and nursing expertise, also limit the adaptability of telemedicine in underdeveloped areas. The growth of telemedicine will hasten the development of technology‐based telehealth solutions, such as real‐time telehealth data collection and remote vital sign monitoring, which will facilitate early diagnosis and treatment commencement. A coordinated shift between virtual and in‐person treatment will be supported by the designation of new workflows improved by artificial intelligence.^[^ ^48^ ^]^ With high bandwidths (10 GB/s), low latency (<1 ms), and high quality of service, fifth‐generation (5G) mobile communication technology has the potential to meet the demands of this digitalized future.^[^ ^49^ ^]^ This will allow for wireless real‐time data transmission in telemedical emergency health care applications, which makes telesurgery available. In China, a doctor has successfully performed radical cystectomy on a bladder cancer patient who was 3000 km away through telemedicine. Additionally, from March to October 2021, 29 patients from eight primary hospitals underwent remote robot‐assisted laparoscopic radical nephrectomy procedures in a Qingdao tertiary hospital, using a surgeon‐controlled surgical robot.^[^ ^50^ ^]^ They found that patients with kidney malignancies have a safe and practical therapeutic option in telesurgery with 5G technology. There were only 200 ms of total latency between the distant location and the operating rooms where the surgery was being performed.^[^ ^50^ ^]^ However, in addition to the surgery itself, anesthesia techniques and postoperative care teams are equally important for the prognosis of patients undergoing such major surgeries. Strengthening telemedicine‐related perioperative management is an unavoidable issue in the process of addressing telemedicine adaptability in the future. Thirdly, the workflow of telemedicine is different from that of a traditional in‐person medical visit, which makes it challenging for some doctors to adapt to the telemedicine working environment and ensure work efficiency and medical quality. The imbalance between the rapidly developing telemedicine and the inadequate adaptability of these medical workers may lead to varying degrees of psychological disorders and even occupational anxiety. Additionally, the reconstruction of communication and trust between doctors and patients brought about by telemedicine is also a crucial challenge for the development of telemedicine. Compared to an in‐person medical visit, telemedicine lacks humanistic care to some extent, and patients feel less emotional connection. Against the backdrop of patients’ low confidence in telemedicine and the general tension between doctors and patients in China, if medical errors or accidents occur in the process of telemedicine, will it further intensify the conflict and contradiction between doctors and patients in China? This is also a question worth considering.

It is indisputable that healthcare professionals and patients exhibit various levels of adaptability towards telemedicine. While medical practitioners possess superior medical knowledge and skills, enabling them to effortlessly acclimate to and master remote medical technologies, patients may encounter different degrees of difficulty in utilizing remote medical technologies, attributable to various factors such as age, education level, and geographical location.^[^ ^12^ ^]^ To address this, it is crucial to implement diverse strategies and measures to optimize the usability and reliability of telemedicine. Healthcare professionals necessitate additional training and support to enable them to utilize remote medical technologies competently, thereby enhancing the quality of diagnosis and treatment. Patients, on the other hand, require more user‐friendly and accessible remote medical platforms and tools, complemented by appropriate guidance and education to augment their capacity and confidence in utilizing remote medical care. Long‐term patient satisfaction with this new care delivery model will be influenced by persistent concerns about the safety of receiving care in‐person as well as practical issues with traveling to destination centers.^[^ ^48^ ^]^ Patients with chronic diseases must frequently interact with the healthcare system, which can be inconvenient for them and result in higher medical expenses, missed visits, and noncompliance with prescribed treatment regimens.^[^ ^48^ ^]^ Maintaining and improving these interactions in ways that patients will find more agreeable is made possible via telemedicine. Meanwhile, we also need to pay attention to the psychological care and counseling for both doctors and patients to relieve the anxiety caused by medical “snack community” and telemedicine. It is highly anticipated that telemedicine will be more closely related to the health care system in the future, because it adds another level to the delivery of effective, cost‐effective healthcare that benefits both patients and health care systems. However, it is important to address the adverse effects of telemedicine and therefore reduce the formation of a telemedicine‐induced medical “snack community” to provide better high‐quality medical care and education. Governments, medical institutions, and technology companies should proactively undertake initiatives to enhance the technological, policy, and service aspects of telemedicine, facilitate its widespread adoption and development, and benefit more medical workers and patients.

## 4 PARTNERS HEALTHCARE SYSTEM (PHS) AND TELEMEDICINE

**FIGURE 1:**
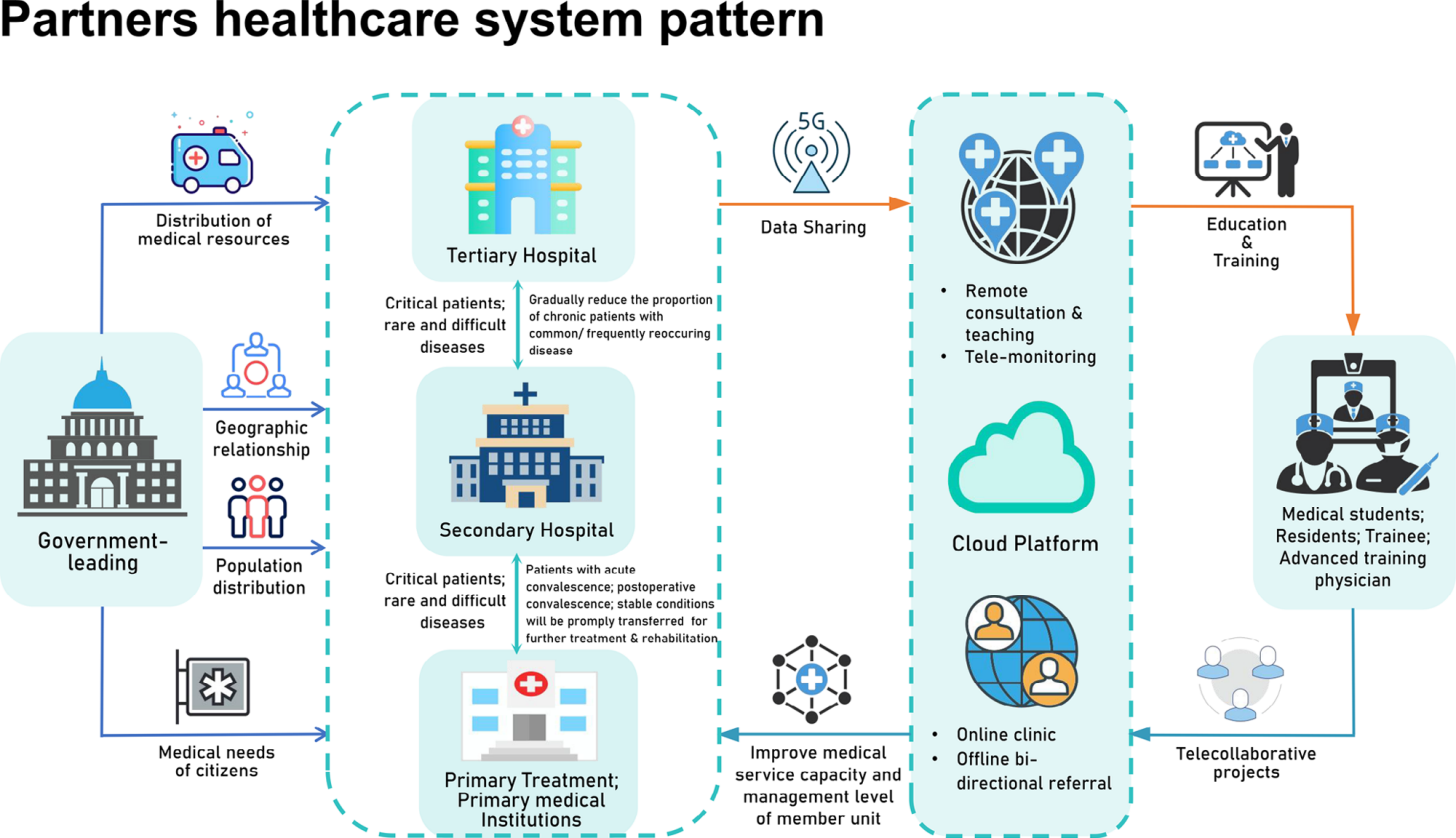
Partners healthcare system pattern.

Founded in 1994, Partners HealthCare System is a $4 billion integrated delivery network located in Boston, Massachusetts.^[^ ^51^ ^]^ Partners is made up of eleven facilities: community and specialty hospitals, academic medical centers (Mass General Hospital and Brigham and Women's Hospital), and a network of over 1000 primary care clinicians who are owned and affiliated.^[^ ^51^ ^]^ The computer physician order entry apps and other clinical information systems at the academic centers are created in‐house. Additionally, Partners’ widely used outpatient electronic medical record, called the Longitudinal Medical Record, was created internally.^[^ ^51^ ^]^ Thus, PHS refers to an intergradation of regional medical resources, usually composed of tertiary, secondary and community hospitals and in a region. Similar patterns could be seen in other countries in order to optimize medical resources and serve patients better. Chinese PHS was proposed over 10 years ago and has been developed rapidly in the past three years, with over 80% of medical organizations involved nationwide (Figure 1). Despite some achievements made currently, Chinese PHS could not be considered as real success. This might be caused by the following reasons: (1) inconsistent ownership; (2) an imperfect hierarchical medical system; (3) a unified electronic system; and (4) a lack of standardized treatment guidelines in clinical practice. In this case, telemedicine based on advanced communication technologies and tiered performance appraisal systems could greatly contribute to the progress of PHS, and this in turn improves the development of telemedicine itself.

PHS with enough bandwidth, especially 5G technology, could optimize the effect of telemedicine in medical practice and education. With the framework of PHS, telemedicine could more effectively upgrade the professional perception and skills of local medical workers and improve patients’ recognition of telemedicine. Additionally, with the help of PHS and real‐time telemedicine technologies, the inconsistence of doctor‐patient telemedicine adaptability was decreased, and more close communications among patients, medical students and practitioners from different hospitals might contribute to the improvement of medical service, an increased sense of professional achievement and more happiness indexes, which further decreases the number of medical “snack community” and leads to an improved telemedicine‐medical “snack community”‐PHS ecosystem. For example, medical students and local medical staff even patients, can learn cutting‐edge expertise from senior experts and academic conferences in superior hospitals. In addition, this collaboration could lead to the construction of disease‐specific cohorts to better understand disease features and healthy management in the Chinese population. Thus, tiered performance appraisal systems are necessary because local medical staff are disadvantaged in all aspects. It is inevitable that PHS will be the presentative of multidisciplinary medical care and education systems now or even in the future. Medical alliances and telemedicine complement each other and jointly promote the reform of medical care and medical education. To achieve this purpose, consistent ownership, an electronic system, standardized treatment guidelines, and armamentarium, shared treatment information, and fair and equal payment are needed to be addressed. Contingency plans need to be prepared at all times since we currently rely too much on electronic systems. We have to assert that the constructions of infrastructure, medical education, and expertise of medical workers are fundamental since PHS and telemedicine are only auxiliary tools to facilitate daily work.

## 5 PERSPECTIVE AND CONCLUSION

**FIGURE 2:**
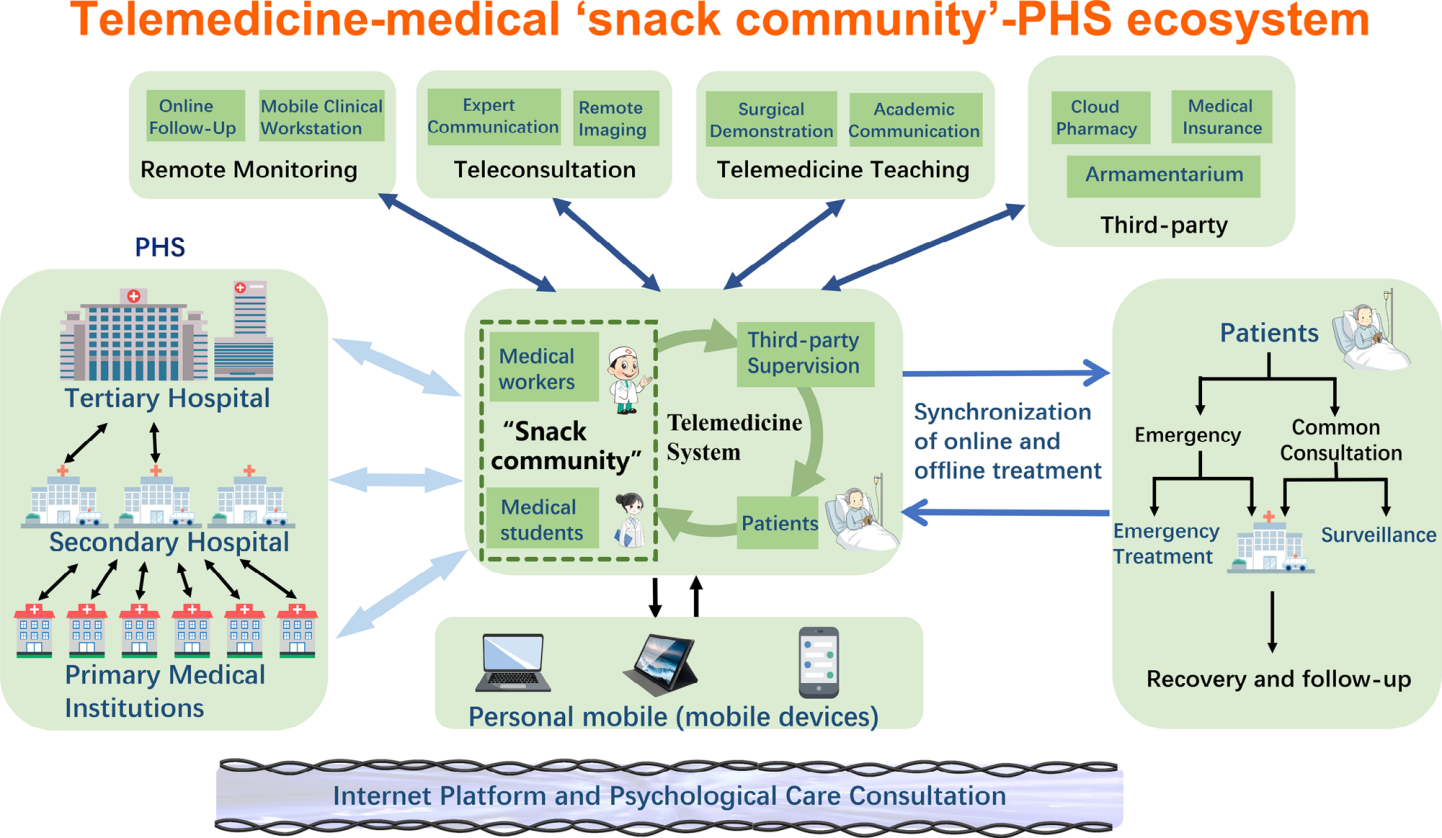
Telemedicine‐medical “snack community”‐partners healthcare system (PHS) ecosystem.

In our opinion, telemedicine plays an important role as a counterbalance in the telemedicine‐medical “snack community”‐PHS ecosystem (Figure 2), which composes a new era of medical patterns in modern society, especially for those rat‐race countries. This phenomenon indicates the plight during the transformation of traditional culture into sack culture in modern society and is thought‐provoking. Thus, we need to slow down, and the support of regulations made by the government and third‐party supervision are prerequisites for the purpose of focusing on the medical education and fitness of medical practitioners and patients. However, we have to proclaim that the constructions of infrastructure, medical education and expertise of medical workers are far more important, as telemedicine can only be used as an auxiliary.

## AUTHOR CONTRIBUTIONS

Dechao Feng, Xu Shi, and Jie Wang proposed the project and wrote the manuscript. Jie Wang, Liying Zhang, and Yuhan Xiao draw the figures. Ruicheng Wu and Dengxiong Li conducted resource seeking. Dechao Feng and Ping Han supervised the project and interpreted the data. All authors reviewed and edited the manuscript.

## CONFLICT OF INTEREST STATEMENT

The authors declare no conflicts of interest.
